# Superimposing Pre-Cranioplasty on Pre-Craniectomy Images to Gauge Feasibility of Early Cranioplasty: A Proof of Concept

**DOI:** 10.1089/neur.2022.0033

**Published:** 2022-08-22

**Authors:** Yu-ying Wu, Han-Jung Chen, Kang Lu, I-Fan Lin

**Affiliations:** ^1^ School of Medicine, College of Medicine, I-Shou University, Kaohsiung, Taiwan.; ^2^ Department of Neurosurgery, E-Da Hospital, Kaohsiung, Taiwan.; ^3^ Division of Infectious Diseases, Department of Internal Medicine, E-Da Hospital, Kaohsiung, Taiwan.

**Keywords:** decompressive craniectomy, early cranioplasty, enhanced recovery after surgery, neuroimaging

## Abstract

Cranioplasty to reconstruct a skull defect after a decompressive craniectomy (DC) is a common neurosurgical procedure. However, cranioplasty is associated with relatively high complication rates, with optimal timing from craniectomy to cranioplasty remaining a controversial matter. Recent studies demonstrated early cranioplasty with appropriate risk mitigation to be a viable option with many clinical advantages, propelling the advocacy for cranioplasty as soon as brain swelling resolves. We report on a 33-year-old male with traumatic brain injury who received an early cranioplasty, 18 days post-DC. The extent of adequate brain swelling resolution was determined by superimposing selected pre-cranioplasty computed tomography (CT) images onto corresponding pre-craniectomy CT images. By ensuring all brain matter lies within the outer table of the skull in superimposed brain images, the extent of brain swelling resolution could be determined reliably and the feasibility of cranioplasty can be assessed objectively.

## Introduction

Therapeutic decompressive craniectomy (DC) for traumatic brain injury (TBI) is regarded as a potential life-saving procedure in patients suffering from increased intracranial pressure (ICP) refractory to medical therapy.^1^ DC involves removing part of the skull until eventual reconstruction using autologous bone grafts or artificial materials, known as cranioplasty (CP).^2^

CP has various benefits, including mechanical protection of the brain, restoration of esthesis, improving cerebrospinal fluid dynamics, and promoting recovery of cortical and subcortical neurological functions.^3^ However, CP is long recognized to be associated with relatively high complication rates, ranging from 15% to 35%.^4^ The timing to CP is under continuous debate to strive for the optimal balance between maximal benefits brought by early CP and minimal risk of complications associated with the procedure.^5^ Literature suggests that CP between 15 and 30 days minimizes the risk of infection, seizure, and autologous bone flap resorption while still minimizing the time the patient, who underwent DC, will spend with a large skull bone defect.^4^

Although recent studies have pushed for early CP,^6^ the feasibility of CP lies in the prerequisite that brain swelling has resolved enough, generating sufficient space for CP to be performable. The current consensus that CP should be performed “as soon as brain edema had normalized”^2^ was generally left open to individual experience and clinical judgments. This case report aimed to present a scientific method to define the aforementioned resolution of brain swelling, using computed tomography (CT) images as objective evidence to push the agenda for the earliest CP possible.

The patient in this case report consented to publishing his deidentified medical information and images. This study was approved as exempt from review by the institutional review board.

## Case Report

**FIG. 1.**
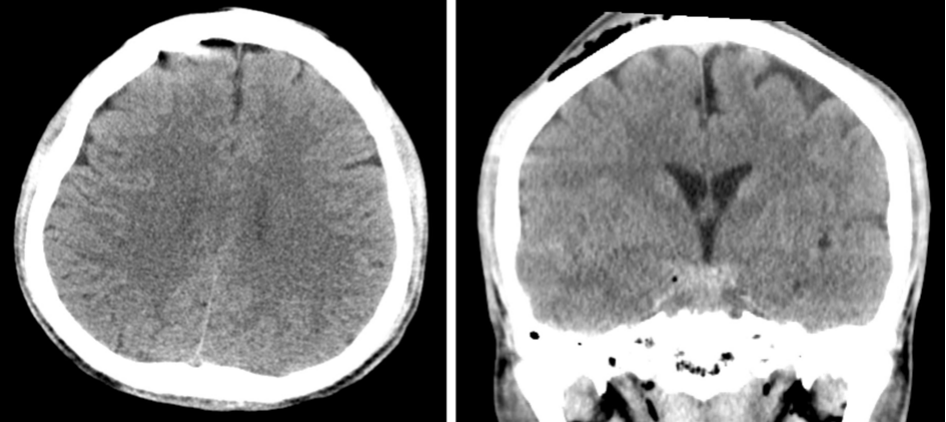
Initial CT of the head. Left, axial view; right, coronal view shows subarachnoid hemorrhage. Lack of midline shift is noted. CT, computed tomography.

**FIG. 2.**
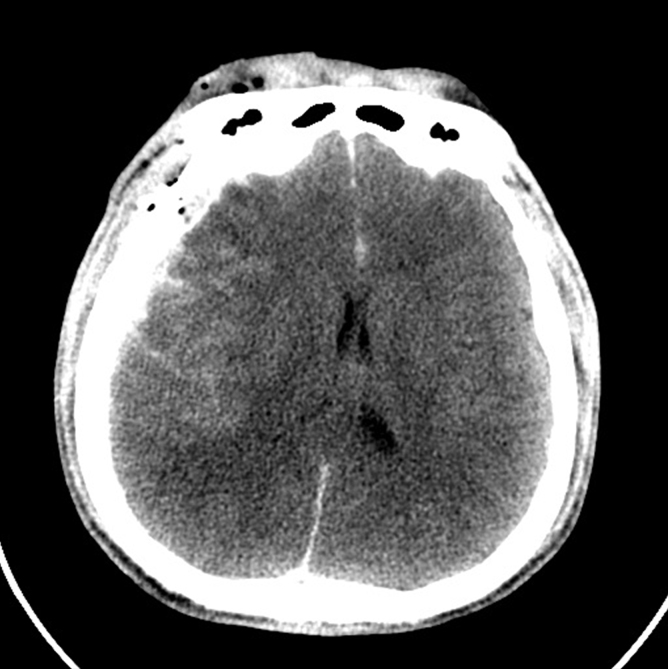
CT of the head before decompressive craniectomy shows right subdural hematoma and increased intracranial pressure with midline shift up to 8 mm. CT, computed tomography.

This was a 33-year-old male without any chronic underlying disease. He was involved in a high-speed motorcycle collision accident, causing TBI, right facial fractures, and a 5-cm open laceration across the right eyebrow. He was sent to our emergency department (ED) within half an hour. He displayed a brief period of a lucid interval. His Glasgow Coma Scale (GCS) dropped from 15 to 9 after 1 h of observation in the ED. Figure 1 shows the axial and coronal views of the patient's initial brain CT, respectively, upon arrival in the ED. Figure 2 shows the follow-up brain CT at 1 h, revealing significant brain swelling and right subdural hematoma progression, causing a brain midline shift up to 8 mm. After a discussion with the patient's family and shared decision making, an emergent DC was performed.

### Decompressive craniectomy

During the operation, severe brain swelling rendered replacing the patient's cranial bone flap impossible. Consequently, the bone flap was sent to the tissue bank freezer for cryopreservation. An ICP monitoring probe (Medtronic, Dublin, Ireland) was placed into the patient's brain parenchyma. The post-operative ICP was 15 mm Hg, and the patient was transferred into an intensive care unit (ICU) for close observation. The patient's GCS recovered to 15 in the ICU, and he was transferred to the surgical ward 5 days later. His ICP gradually decreased to 10 mm Hg on day 7 after DC, and the ICP monitoring probe was removed.

### Superimposing imaging method

**FIG. 3.**
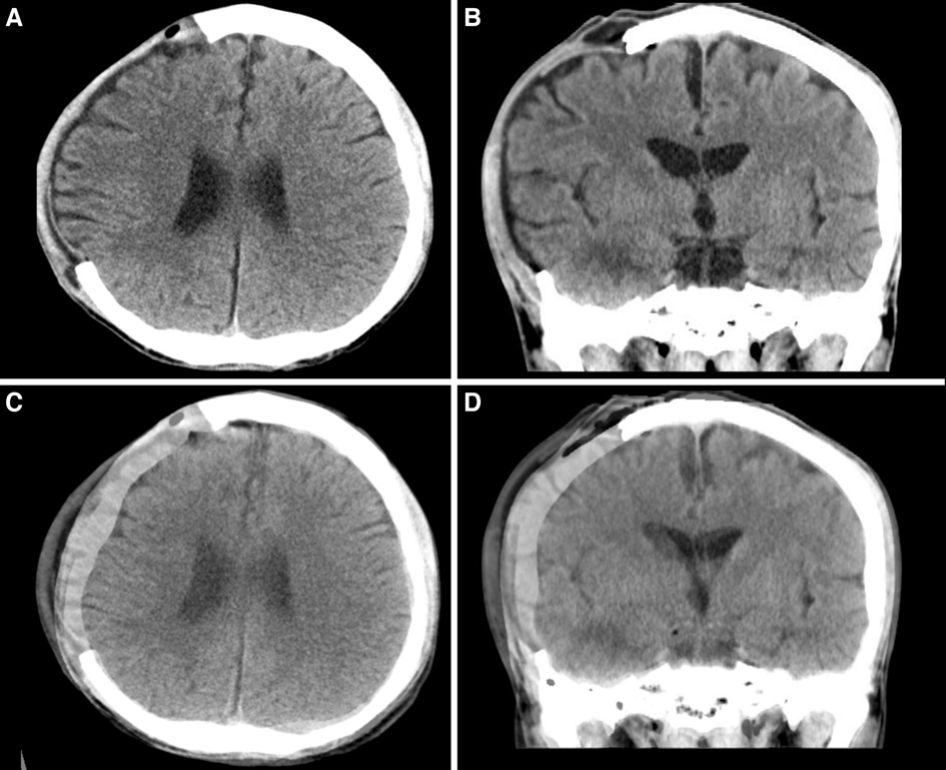
CT of the head. (**A**) Axial and (**B**) coronal views of the greatest diameters of skull defect before CP. Presence of interhemispheric hygroma is noted. (**C**) Axial and (**D**) coronal views of the pre-CP images adjusted to 50% transparency superimposed onto corresponding pre-DC images (Fig. 1). All brain matter lies within the outer table of the skull. CP, cranioplasty; CT, computed tomography; DC, decompressive craniectomy.

On day 17 after DC, his skull defect's bone edge first showed partial physical visibility under the scalp. A pre-CP brain CT was obtained to evaluate the extent of brain swelling resolution and gauge the feasibility of CP. The CT slices of axial and coronal views representing the largest diameters of skull defects were extracted, as shown in Figure 3 (panels A and B, respectively). The corresponding CT slices from the pre-DC brain CT were extracted, as represented in Figure 1. Then, after adjusting the image transparency of pre-CP images to 50%, they were superimposed onto Figure 1, as shown in Figure 3 (panels C and D, accordingly). The superimposing method was performed using Google Slides, as demonstrated in Supplementary Video S1. Because the entirety of brain matter lay within the outer table of the skull, brain swelling resolution was considered adequate and CP was deemed feasible. CP was performed the day after pre-CP brain CT evaluation, which was 18 days after DC. The patient consented to the procedure.

### Early cranioplasty

CP was performed using the autologous bone flap that was stored in cryopreservation. Because early CP was done, no adhesion was encountered during the operation. The scalp was reflected with relative ease, and the autologous bone flap was replaced into the skull defect site without difficulty. The bone flap was anchored with one silk line at each of its four borders. One subgaleal drain was placed, and the surgical wound was closed in layers. Total procedural time was 42 min, and estimated blood loss was 50 mL.

A few intraoperative techniques could be used to facilitate a smooth CP procedure. First, the patient was positioned in a semi-Fowler's position to reduce brain swelling.^7^ Second, a 300-mL bolus of mannitol was given intravenously at the start of CP to reduce brain swelling further^8^ and generate more space for the autologous bone flap placement. Third, anesthesiologists avoided using anesthesia agents that increase ICP, such as nitrous oxide and isoflurane.^9^ Fourth, a hinge craniotomy (HC) could be applied when necessary.^10^ However, HC was unnecessary in this case because the bone flap could be aligned in its desired position with ease.

### Outcome

**FIG. 4.**
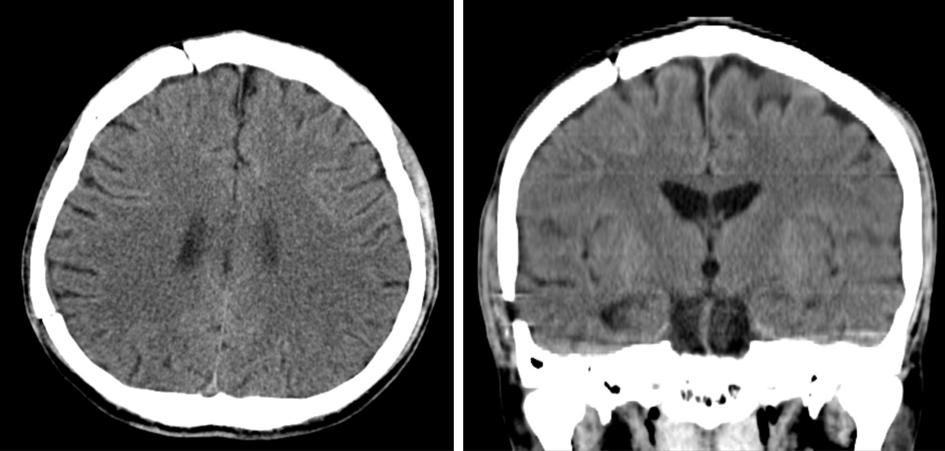
CT of the head, 40 days after cranioplasty. Left, axial view; right: coronal view. CT, computed tomography.

The patient recovered from the CP without any neurological symptoms and his neurocognitive function improved. The subgaleal drain was removed 2 days after CP, and the patient was discharged 8 days after CP. Forty days after CP, during a follow-up in outpatient clinics, a brain CT was obtained (Fig. 4). The patient made an excellent recovery, able to carry out all usual activities (Modified Rankin Scale = 1).

## Discussion

Large bodies of literature have shown the benefits of early CP, leading to the proposition to perform CP “as soon as brain swelling resolves on CT scan.”^2^ This begs the question to define “brain swelling resolution” in a scientific manner that can be utilized in clinical settings. This is the first case report to discuss the issue objectively, describing an imaging technique to gauge the extent of brain swelling resolution to determine CP feasibility. The method is simple and straightforward, as described above and shown in Supplementary Video S1, by superimposing pre-CP images onto pre-DC images to determine whether all brain matter lies within the outer table of the skull.

Instinctively, the inner table of the skull should be the safest anatomical reference margin to ensure no increased ICP after CP. However, the outer table of the patient's skull was chosen in this proof of concept to promote the earliest CP possible. Moreover, numerous studies have demonstrated the clinical usefulness of HC.^11^ The limitation of the HC revolves around whether sufficient extracranial brain expansion volume will be achieved and whether the patient will require DC later on.^11^ Using the superimposing images method described in this case report, the entire brain volume would undoubtedly be within the space confined by a HC without causing increased ICP, negating all the main concerns in HC. On a side note, if artificial materials were used for CP, such as custom-made three-dimensional printed synthetic alternatives, the outer table would be used as a reconstruction scaffold,^12^ rendering the concept described in this case report to be an even more relevant gauge.

Regardless of the many advantages of an early CP, one simply cannot overlook the staggering associated risks involved.^4^ There were still substantial studies warning of the higher complication rates of early CP,^13,14^ suggesting CP to be performed between 3 and 6 months.^15^ Evidently, an early CP is not universally appropriate. Nevertheless, an early CP was indicated in this case because of the development of interhemispheric hygroma (Fig. 3A,B), where studies have shown that >80% will progress to hydrocephalus without intervention.^16^ Through this case report, as shown in the follow-up brain CT images (Fig. 4), we have arrested the ventriculomegaly and effectively prevented hydrocephalus by performing an early CP.^5,17^

This case report was specifically chosen to demonstrate the maximum tolerable limit of this superimposing imaging method because the brain matter lined exactly at the outer table of the skull (Fig. 3C,D), making it the best representation of this proposed proof of concept. There were a few other cases, who underwent early CP with good outcomes at our institution, whose superimposed CT images showed brain swelling resolution to a much greater extent. In those cases, traditional “eyeballing” would probably suffice and pre-CP brain CT served as more of an adjuvant gauge. Other imaging criteria could play pertinent roles in gauging CP feasibility, such as brain sulcus widths, midline deviations, presence of intracranial hemorrhage, and computed brain volumes. It goes without saying that the decision to perform a CP cannot be made on radiographical evidence alone; one must take the patient's clinical course, ICP, and neurological status into account.

This case report demonstrated the feasibility of early CP from a space-occupying standpoint using superimposed CT images as direct evidence. Certainly, more studies are needed to demonstrate the safety and added value of this proposed proof of concept. The associated complications with early CP could be devastating. It was hoped that this concept would be helpful in the clinical decision-making process to perform the earliest CP possible without increasing morbidity and mortality. The ongoing debate regarding the best timing to perform a CP is beyond the scope of discussion in this case report. The main purpose of this case report was to present a scientific method to determine the adequacy of brain swelling resolution to gauge the feasibility of CP. In the future, more of such patient-orientated, individualized imaging criteria could be explored.

## Conclusion

The simple and intuitive concept to superimpose pre-cranioplasty images onto pre-craniectomy images proved to be a reliable method to gauge whether brain swelling has resolved enough for a CP to be performed. More such objective clinical criteria are needed to improve the safety of early CP.

## Supplementary Material

Supplemental data

## Abbreviations Used

CP: cranioplasty
CT: computed tomography
DC: decompressive craniectomy
ED: emergency department
GCS: Glasgow Coma Scale
HC: hinge craniotomy
ICP: intracranial pressure
ICU: intensive care unit
TBI: traumatic brain injury
